# An early diagnosis of trichorhinophalangeal syndrome type 1: a case report and a review of literature

**DOI:** 10.1186/s13052-018-0580-z

**Published:** 2018-11-20

**Authors:** Giulia Trippella, Paolo Lionetti, Sara Naldini, Francesca Peluso, Matteo Della Monica, Stefano Stagi

**Affiliations:** 1 Department of Mother and Child’s Health, Meyer University Children’s Hospital, University of Florence, Viale Pieraccini 34, 50100 Florence, Italy; 2 Division of Pediatric Gastroenterology and Nutrition, Meyer University Children’s Hospital, University of Florence, Florence, Italy; 3 Division of Pediatric Gastroenterology and Nutrition, Meyer University Children’s Hospital, Florence, Italy; 4 Medical Genetics Unit, Department of Biomedical Experimental and Clinical Sciences “Mario Serio”, University of Florence, Florence, Italy; 5 Medical Genetics Unit, Department of Biomedical Experimental and Clinical Sciences “Mario Serio”, Florence, Italy; 6 Division of Pediatric Endocrinology, Meyer University Children’s Hospital, University of Florence, Florence, Italy

**Keywords:** Trichorhinophalangeal syndrome, TRPS, Growth retardation, Sparse hair, Bulbous nasal tip, Short fingers

## Abstract

**Background:**

Trichorhinophalangeal syndrome (TRPS) is a rare autosomal dominant disorder caused by defects involving the *TRPS1* gene. It exhibits distinctive craniofacial, ectodermal and skeletal abnormalities, such as sparse hair, bulbous nasal tip and short deformed fingers, with extremely variable expressivity.

**Case presentation:**

We report the case of a 17 months old girl, who presented growth retardation and dysmorphic features. Postnatal growth was always below − 2 Standard Deviation for both weight and length and physical examination revealed relative macrocephaly, sparse hair, bulbous nasal tip, thin upper lip, protruding ears, prominent forehead, small jaw, and short hands and feet. Patient’s mother shared the same facial features, and presented sparse hair and small hands. The maternal grandfather and two uncles presented short stature, bulbous nasal tip, thin hair, and premature alopecia. Molecular analysis of *TRPS1* gene showed a heterozygous c.2086C > T;(p.Arg696Ter) mutation both in the patient and her mother, confirming the diagnosis of TRPS, type I.

**Conclusions:**

Clinical phenotype of TRPS can be subtle and the syndrome often remains undiagnosed. A comprehensive clinical examination and an exhaustive family history are crucial to reach the correct diagnosis, which is essential to perform adequate follow-up and timely therapeutic procedures.

## Introduction

Trichorhinophalangeal syndrome (TRPS) is a rare disorder characterized by distinctive craniofacial and skeletal abnormalities, first described in 1966 by Giedion, who named the syndrome on the basis of the three main features: sparse hair, bulbous nasal tip and short deformed fingers [1].

TRPS is caused by a pathogenic variant of the *TRPS1* gene, inherited in an autosomal dominant manner, with high penetrance and variable expressivity. The disease could also be a result of a de novo mutation [2].

Three subtypes of TRPS are described: TRPS I, TRPS II and TRPS III, differing in clinical characteristics and pattern of mutation in the *TRPS1* gene. All three subtypes of TRPS presented distinctive craniofacial features, ectodermal and skeletal anomalies. Craniofacial features include bulbous tip of the nose, long flat philtrum, thin upper lip with vermilion border and protruding ears. Ectodermal anomalies consist of slowly growing and sparse scalp hair, with receded medio-occipital hairline, medially thick and laterally thin eyebrows, dystrophic nails and dental anomalies, such as supernumerary teeth. Skeletal anomalies include short phalanges and short metacarpals (mild to severe brachydactyly), ulnar or radial deviation of the fingers, cone-shaped epiphyses, hip malformations and short stature [3]. TRPS II is characterized by multiple exostoses and an increased risk of mild-to-moderate intellectual disability. TRPS III is considered an extreme of the clinical spectrum of TRPS I, with more severe brachydactyly due to short metacarpals, and severe short stature.

Clinical presentation could vary widely, from simple change in the phalanges to low bone mineral density with high risk of fragility fractures. Furthermore, there are reports of cardiac and neurologic abnormalities [5].

The exact prevalence of TRPS is not known. Around 100 cases of TRPS I and TRPS III and 100 cases of TRPS II were published until June 2017. However, given the widely variable manifestations, many cases of TRPS probably remain undiagnosed [4].

## Case report

We describe the case of a 13 months’ girl, brought to the gastroenterology department of a third level pediatric hospital because of failure to thrive.

The patient was the only child born to unrelated parents. During pregnancy, first-trimester combined test showed abnormal values of pregnancy-associated plasma protein (PAPP-A) and free beta human chorionic gonadotropin (β-hCG), while ultrasound test for fetal nuchal translucency resulted normal. Chorionic villus sampling and cell-free fetal DNA analyses were performed at 12 weeks’ gestation (WG) and showed no evidence of chromosomal disorders.

**Fig. 1:**
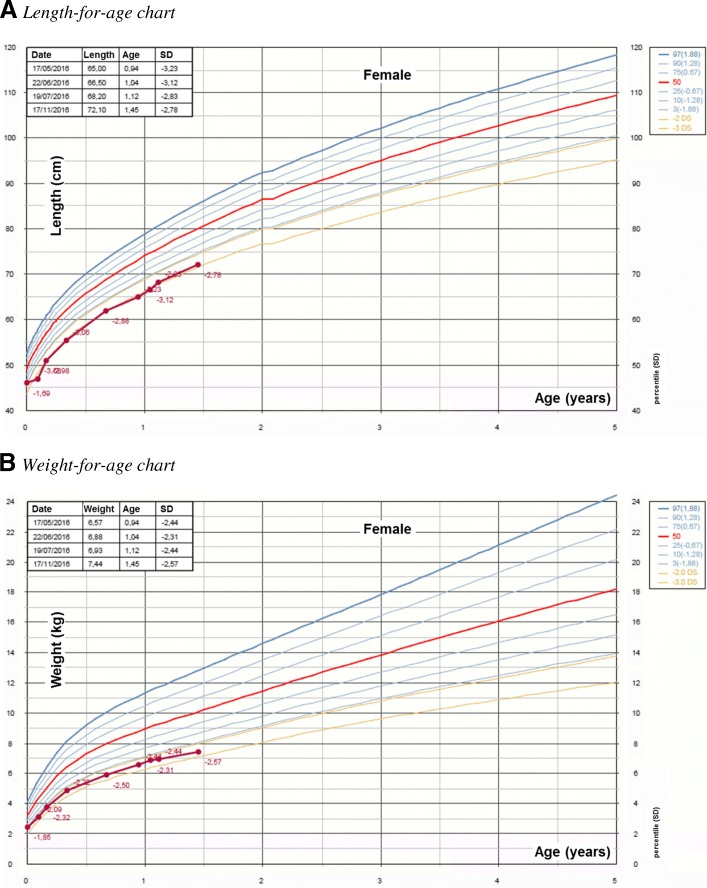
Patient’s growth charts (WHO growth charts for girls ages 0 to 5 years of age). **a** Length-for-age chart. **b** Weight-for-age chart

During the sixth month of gestation, prenatal ultrasound revealed intrauterine growth retardation, hence the mother was treated with low-molecular-weight heparin. At 38 WG and 6 days labor was induced, due to the onset of pre-eclampsia. At birth, the child was small for gestational age. Birth-weight was 2452 g (− 1.85 Standard Deviation, SD), length 46 cm (− 1.69 SD) and occipito-frontal head circumference (OFC) 34 cm (− 1.22 SD). Apgar score was 8^I^ to 9^V^. The child presented a talipes valgus of both feet, hence was treated with taping for three months. Neuromotor development was normal, nevertheless the child presented speech delay. Postnatal growth was regular, but always below − 2 SD for both weigh and length (Fig. 1 a, b).

Because of the poor growth the patient underwent several medical assessments. An endocrinological evaluation was performed at the age of 8 months. Laboratory tests, including hemochrome, thyroid function, cortisol, Insulin-like Growth Factor I (IGF1) and 25-OH Vitamin D3 dosage, resulted normal; a radiograph of the left hand revealed delayed bone maturation. Furthermore, CGH-array analysis was performed; the test excluded the presence of DNA microdeletion/microduplication.

**Fig. 2:**
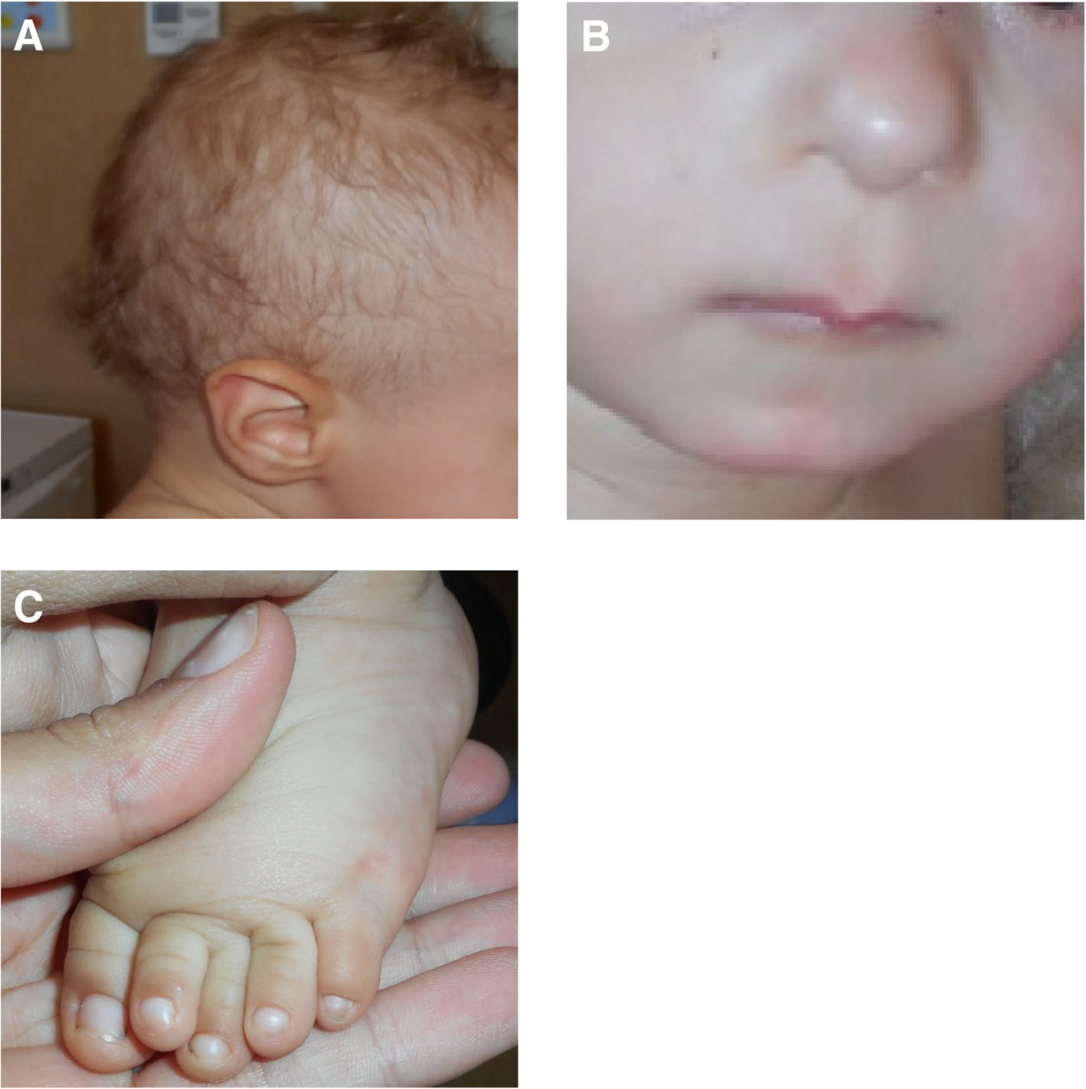
Typical phenotipic features. **a** Fine and sparse hair. **b** Bulbous nose, long philtrum, thin upper lip. **c** Short foot withbrachydactyly of the 2nd finger (**c**)

The patient has come to the gastroenterology department of our hospital at the age of 13 months, because of growth retardation and behavioral feeding disorders. The parents reported limited appetite and selective intake of food. Weight was 6.930 kg (− 2.38 SD), length was 68.4 cm (− 2.64 SD), OFC was 45.8 cm (+ 0.34 SD). Physical examination revealed peculiar characteristics: sparse hair, bulbous nasal tip, thin upper lip, protruding ears, prominent forehead, small jaw, and short hands and feet (Fig. 2 a, b, c). Advise was given to the family regarding correct feeding techniques. Furthermore, the patient was evaluated by the genetics unit of our hospital. Considering the poor growth, the relative macrocephaly and facial features, Silver-Russel syndrome was initially suspected, but deletion/duplication analysis of 11p15 and methylation analysis of DMR1 (H19) and DMR2 (KCNQ1OT1) regions showed no anomalies.

**Fig. 3:**
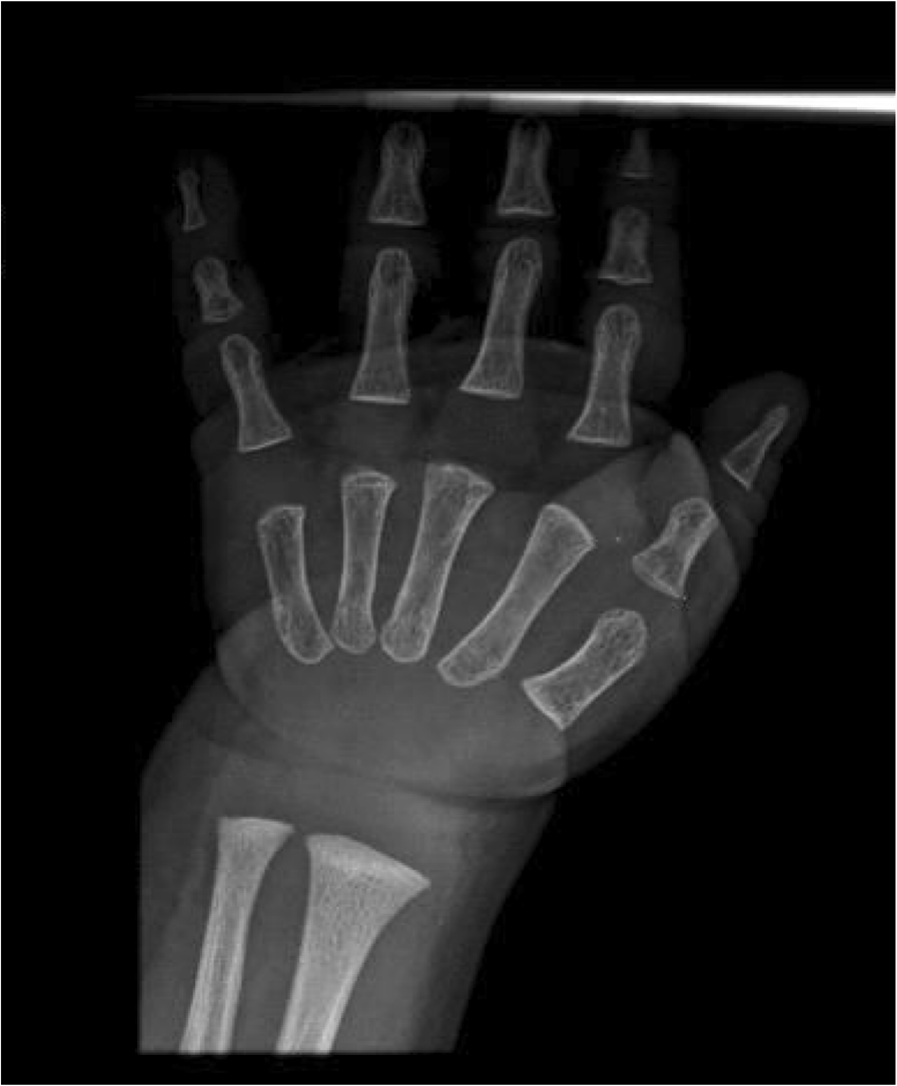
Radiograph of patient’s left hand. Delayed bone maturation, cone-shape appearance of distal phalanges. (We could not perform a morphologic radiographic study due to the lack of images for third and fourth distal phalanges)

**Fig. 4:**
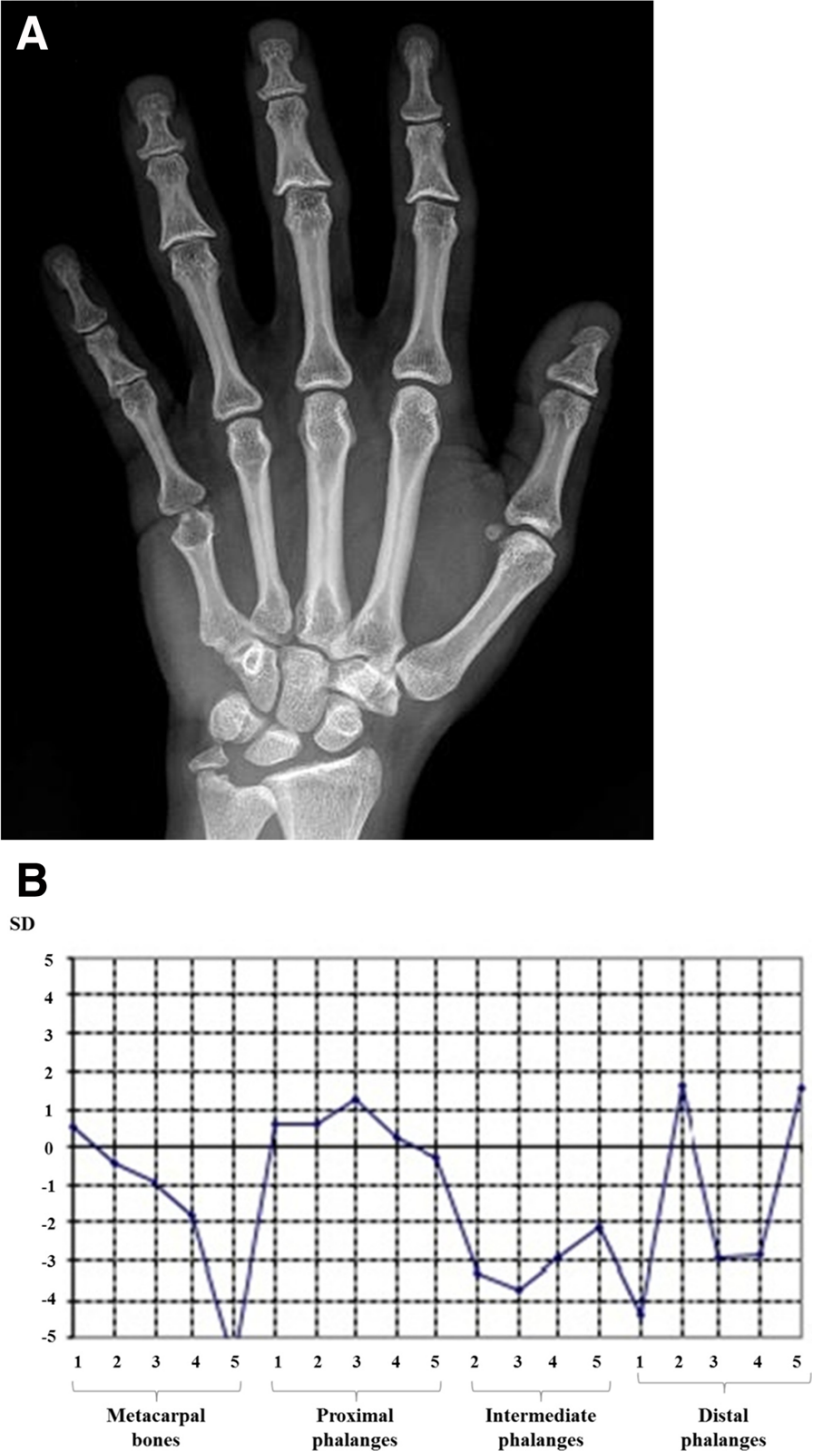
Radiograph of the mother’s left hand with morphologic study. **a** Cone-shaped epiphyses of middle phalanges, clinodactyly of 2nd finger, and brachymetacarpalia of 5th metacarpal. **b** Morphologic radiographic study: several bone segments are below − 2. Standard Deviation (SD): particularly, the fifth metacarpal bone is below − 5 SD

On the occasion of a check-up, at the age of 17 months, we noticed that patient’s mother shared the same facial features of the baby, and presented sparse hair and small hands. She was 158 cm tall. Extending the family history, we found out that the maternal grandfather and the two uncles presented short stature, had the same bulbous nasal tip and thin hair, furthermore they developed alopecia before the age of 20. This information led us to suspect trichorhinophalangeal syndrome, inherited from the maternal lineage, hence we decided to reevaluate the radiograph of the left hand previously made, which revealed a cone-shape appearance of the last phalanges (Fig. 3). Furthermore, we perform a radiograph of the hands of patient’s mother, which showed cone-shaped epiphyses of middle phalanges of 2nd, 3rd and 4th fingers bilaterally, clinodactyly of the 2nd finger bilaterally, brachymetacarpalia of 5th metacarpal bilaterally, with dysmorphic aspect of distal epiphyses (Fig. 4 a, b). All of these radiographic features were consistent with the hypothesis of trichorhinophalangeal syndrome.

### Molecular study

DNA was extracted from blood samples of both patient and patient’s mother. Direct sequencing of PCR products was performed, covering all protein-coding exons of *TRPS1* gene and the consecutive intronic regions. Molecular analysis of *TRPS1* gene showed a heterozygous c.2086C > T;(p.Arg696Ter) mutation in the patient and her mother, allowing us to confirm the diagnosis of TRPS I.

## Discussion

This report reflects the complexity of identifying a patient with TRPS. This disease is rare and usually not well-known by doctors; it has extremely variable expression, with clinical phenotypes that might be fairly mild. Therefore, TRPS often remains undiagnosed. This entails the risk that patients undergo numerous unnecessary investigations before reaching the correct diagnosis, which is often made when a more severely affected family member presents with the classic phenotype.

In the described case, at least 4 additional family members had never received a diagnosis. Our patient had undergone numerous medical assessments and laboratory tests, but only a careful medical history and accurate examination allowed the diagnosis, along with the knowledge of the syndrome.

Our patient was diagnosed at 17 months old, earlier than most of the cases described in literature. Although there is currently no curative treatment for TRPS, a correct diagnosis is essential to perform all the supportive care that the patient would need. Particularly, timely orthopedic procedures can correct functional disability and physiotherapy can prevent secondary joint degeneration and chronic arthralgias. Furthermore, an early diagnosis allows a strict control of the growth pattern and an investigation of GH production. In case of poor growth and GH deficiency, a replacement therapy can be offered. Mutation analysis extended to the other family members allows identification of carriers and subsequent genetic counseling.

The molecular study of our patient revealed a pathogenic mutation on *TRPS1* gene. This mutation, c.2086C > T, was previously described by Dias et al., in a girl with a sporadic case of TRPS I. In that case, two mutations were found in the TRPS1 gene, both in heterozygosity, c.1198C > T (p.Gln400X) and c.2086C > T (p.Arg696X). The patient presented a pear-shaped nose, a long philtrum, thin lips and a broad forehead. At four years old, her weight was in 5th, height in 10th, and head circumference in 75th percentiles. The same patient was described by Marques et al.: at ten years old she presented short stature for her age (− 2.03 SD) with a reduced growth velocity (− 4.07 SD) and a bone age of 4.5 years. GH stimulation test was performed, showing GH deficiency. Therefore GH replacement therapy was undertaken, obtaining a satisfying catch-up growth [6, 7].

The case described by Dias et al. and Marques et al. presented two mutations on *TRPS1* gene. Both of them had not been previously described, hence, we cannot know which one contributed the most to the clinical pattern of the patient. Furthermore, the authors could not differentiate whether the two mutations were coexisting in the same allele or in different alleles. Since the patient manifested the typical phenotype of heterozygous TRPS I patients, the authors presumed that the two mutations coexisted in the same allele, behaving as a single mutation. In that case, it is possible that one of the two mutations was not pathogenic.

Clinical features of the patient described by Dias et al. and Marques et al appeared comparable with our patient, therefore we can speculate that the mutation c.2086C > T (p.Arg696X) could be responsible of the phenotype. Additional studies could be helpful to clarify the pathogenic role of these two gene mutations.

Considering the similarities within the described cases, if growth pattern of our patient will remain below the normal range, it would be appropriate to perform a GH stimulation test and a replacement therapy in case of GH deficiency. Given the good response to the replacement therapy described by Marques et al.*,* we are confident to obtain a similar outcome.

### Trichorhinophalangeal syndrome

#### Genetics

*TRPS1* gene is located on chromosomal band 8q24.1, it encodes a transcription factor for a zinc-fingerprotein and represents a candidate gene for bone homeostasis regulation, as it seems to be involved in regulation of bone perichondrium mineralization and proliferation and apoptosis of chondrocytes [6, 9].

TRPS I is caused by heterozygous pathogenic variant of the *TRPS1* gene, which leads to haploinsufficiency of *TRPS1*. To date, more than 130 pathogenic variants in *TRPS1* have been reported [10, 15]; 76 mutations are listed in the public HGMD database (Human Gene Mutation Database; www.hgmd.org), mostly missense and nonsense mutations, or small deletions and insertions within the coding part of *TRPS1* gene [11].

TRPS II, also known as Langer–Giedion syndrome, is considered a contiguous gene syndrome, caused by a larger deletion gene in the long arm of chromosome 8 (8q24.11–13) involving *TRPS1* and *EXT1* genes. The band 8q24.13 contains the *EXT1* gene, which encodes a Golgi localized type 2 transmembrane protein with glycosyltransferase activity. EXT1 seems to have a regulatory effect on longitudinal bone growth and is involved in the development of exostoses. Mental retardation appeared to be correlated with the size of the interstitial 8q deletion [12].

TRPS III represents a variant of TRPS I, several missense mutations of the same gene have been described in patients with TRPS III phenotype [10].

#### Diagnosis

History and physical examination can suggest the diagnosis. Every time a patient shows typical craniofacial, ectodermal and skeletal features, TRPS syndrome should be considered.

Imaging studies, especially of the hands, could be useful. Radiographic features include: cone-shaped epiphyses of the middle phalanges of the hands and feet, present in almost all individuals with TRPS and detectable typically after 2 years of age, when epiphyses are just forming; short metacarpals and short phalanges; clinodactyly. Radiographic studies of pelvis and hip resulted often unspecific, showing hip anomalies such as coxa vara, coxa plana, and coxa magna, Perthes disease-like femoral head changes. Multiple exostoses of long tubular bones are typical of TRPS II [13, 14].

TRPS I definite diagnosis can be established with the identification of a heterozygous pathogenic variant in TRPS1 gene. Sequence analysis of TRPS1 can be undertaken first. If no pathogenic variant is detected, either chromosome microarray analysis (CMA) or gene-targeted deletion/duplication analysis is indicated. Karyotype can be considered to detect an apparently balanced translocation or inversion involving 8q24 [8].

If patient present suggestive findings of TRPS II, a CMA can be performed to identify a contiguous 8q24.11–13 deletion that spans the TRPS1-EXT1 interval.

In patient with TRPS phenotype, severe brachydactyly and severe short stature, TRPS III may be suspected. The identification of a specific missense mutation can support the diagnosis.

Molecular genetic testing is not always required to make the diagnosis, as the phenotype is often noticeable and distinct. However, molecular confirmation can be helpful when the clinical presentation is mild or atypical. Furthermore, a genetic diagnosis extended to the family members, could be useful to clarify the risk of recurrence and to provide counseling about family planning and reproductive options.

#### Management

Management of TRPS is essentially supportive. A multidisciplinary care is beneficial, with at least genetic, auxologic, orthopedic counseling.

A regular auxological assessment is important, with frequent measurement of height and growth velocity. In patients showing a growth pattern below the normal range for age and sex, it is indicated to evaluate the growth hormone (GH) secretion with GH stimulation test, as several cases of GH deficiency have been described. In case of proven GH deficiency, GH substitutive therapy can be considered. Encouraging results have been obtained on growth velocity in a number of cases, however patients with TRPS may present a remarkable impairment of bone status with a reduced bone mass and quality, making them unresponsive to the GH therapy [6, 16–20].

A significant reduction of bone mass and quality, as well as severe osteoporosis, has been reported in several patients with TRPS [17, 21]. An evaluation of bone status with dual X-ray absorptiometry (DXA) could be appropriate. In children, Broadband Ultrasound Attenuation (BUA) could be used as a valid alternative of DXA in the assessment and monitoring of bone status [22].

Patients can develop secondary joint degeneration, potentially involving any joint, but most commonly hips and fingers. Osteoarthritis-like changes affecting the large joints represent the main long-term morbidity associated with TRPS, leading to pain and decreased mobility, starting from adolescence or early adulthood. MR imaging can allow an early evaluation of the joints cartilage status, bringing information about articular consequences and allowing to rule out other diseases or complications, such as hip aseptic necrosis. Physiotherapy could improve joint mobility. Physical exercise should be proposed, while discouraging high-impact or contact sports [14, 23].

Psychological support could be beneficial for individuals who are uncomfortable with their physical appearance.

Genetic counseling should be offered to all families with members affected by TRPS. Particularly, young adults at risk of having child with TRPS could benefit from discussion of potential risks to offspring and explanation of reproductive options.

## Conclusion

Clinical phenotype of TRPS can be subtle and the syndrome often remains undiagnosed. A comprehensive clinical examination and an exhaustive family history are crucial to reach the correct diagnosis, which is essential to perform adequate follow-up and timely therapeutic procedures.
